# Comparison of Various Indices in Identifying Insulin Resistance and Diabetes in Chronic Spinal Cord Injury

**DOI:** 10.3390/jcm10235591

**Published:** 2021-11-28

**Authors:** Gary J. Farkas, Phillip S. Gordon, Nareka Trewick, Ashraf S. Gorgey, David R. Dolbow, Eduard Tiozzo, Arthur S. Berg, David R. Gater

**Affiliations:** 1 Department of Physical Medicine and Rehabilitation, University of Miami Miller School of Medicine, Miami, FL 33136, USA; etiozzo@med.miami.edu (E.T.); dgater@miami.edu (D.R.G.); 2 Hackensack Meridian JFK Johnson Rehabilitation Institute, Edison, NJ 08820, USA; phil.gordon.md@gmail.com; 3 University of Miami Miller School of Medicine, Miami, FL 33136, USA; n.trewick@med.miami.edu; 4 Spinal Cord Injury and Disorders Center, Hunter Holmes McGuire VA Medical Center, Richmond, VA 23249, USA; Ashraf.Gorgey@va.gov; 5 Department of Physical Therapy, William Carey University, Hattiesburg, MI 39401, USA; ddolbow@wmcarey.edu; 6 College of Osteopathic Medicine, William Carey University, Hattiesburg, MI 39401, USA; 7 Department of Public Health Sciences, Penn State College of Medicine, Hershey, PA 17033, USA; asb17@psu.edu; 8 The Miami Project to Cure Paralysis, University of Miami Miller School of Medicine, Miami, FL 33136, USA

**Keywords:** spinal cord injury, intravenous glucose tolerance test, insulin sensitivity, insulin resistance, type 2 diabetes mellitus

## Abstract

The purpose of this screening and diagnostic study was to examine the accord among indices of glucose metabolism, including the Homeostatic Model Assessment for Insulin Resistance (HOMA), HOMA2, Matsuda Index, Quantitative Insulin-sensitivity Check Index (QUICKI), hemoglobin A1C (HbA1C), and fasting plasma glucose (FPG) against intravenous glucose tolerance test-measured insulin sensitivity (Si) in individuals with chronic motor complete SCI. Persons with chronic (≥12-months post-injury) SCI (*n* = 29; 79% men; age 42.2 ± 11.4; body mass index 28.6 ± 6.4 kg/m^2^; C4-T10) were included. Measures were compared using adjusted R^2^ from linear regression models with Akaike information criterion (AIC, a measure of error). QUICKI had the greatest agreement with Si (adjusted R^2^ = 0.463, AIC = 91.1, *p* = 0.0001), followed by HOMA (adjusted R^2^ = 0.378, AIC = 95.4, *p* = 0.0008), HOMA2 (adjusted R^2^ = 0.256, AIC = 99.7, *p* = 0.0030), and the Matsuda Index (adjusted R^2^ = 0.356, AIC = 95.5, *p* = 0.0004). FPG (adjusted R^2^ = 0.056, AIC = 107.5, *p* = 0.1799) and HbA1C (adjusted R^2^ = 0.1, AIC = 106.1, *p* = 0.0975) had poor agreement with Si. While HbA1C and FPG are commonly used for evaluating disorders of glucose metabolism, QUICKI demonstrates the best accord with Si compared to the other measures.

## 1. Introduction

Insulin resistance, or decreased insulin sensitivity (Si), is defined as the decreased responsiveness to the metabolic actions of insulin and the pathophysiological response to insulin-mediated glucose uptake in tissue [1,2]. Insulin resistance is a preceding factor in the development of type 2 diabetes mellitus (T2DM) for persons with and without spinal cord injuries (SCI) [1,3,4]. Duckworth et al. [5] identified that 40% of individuals with SCI were glucose intolerant and had hyperinsulinemia. Gater et al. [6] reported in 473 veterans with SCI that approximately half currently had or were previously diagnosed with T2DM. Lavela et al. [7] identified a greater prevalence of diabetes among SCI veterans than the general population with a stepwise increase following the age of 40. Gater and colleagues [8] recently observed that 32% of 71 persons with chromic motor complete SCI had a fasting plasma glucose (FPG) above 100 mg/dL (denoting prediabetes) or were under treatment for T2DM. Additionally, Cragg et al. [9] and Lai et al. [10] reported greater odds and adjusted hazard ratios, respectively, in persons with SCI compared to nondisabled controls, and Peterson et al. [11] recently calculated an incidence almost double that of persons without SCI. These data underscore that glucose dysregulation is a profound public health issue in the SCI population that warrants universal surveillance.

The most accurate methods available for estimating insulin resistance and sensitivity are the euglycemic hyperinsulinemic clamp (EHIC) and the intravenous glucose tolerance test (IVGTT) [12,13,14]. EHIC is rarely used in clinical practice or in research studies because it is expensive and labor-intensive, requires sophisticated equipment and highly trained personnel, and poses a safety risk for many vulnerable clinical populations, including those with SCI [2,14]. EHIC also uses steady-state insulin levels that may be supraphysiological and can result in a reversal of the normal portal-to-peripheral insulin gradient. The IVGTT, when compared to the EHIC, is favored as it is less expensive, safer, easier to administer, and assesses both peripheral glucose tolerance and insulin responsiveness. It also does not require a steady-state condition or intravenous infusions that require constant adjustment [2]. Unlike the EHIC, information about Si and glucose effectiveness (Sg) can be derived from a single dynamic test [2,15].

Data from the IVGTT are subjected to minimal model analysis using the MINMOD computer program. The program produces an index of Si and Sg based on basal fasting and plasma glucose and insulin data obtained throughout the test [13,16]. The minimal model program is defined by two coupled differential equations with modeled parameters determined during the test. Sg is calculated from one model parameter and is defined as the ability of glucose per se to promote its own disposal while inhibiting hepatic glucose production in the absence of an incremental insulin effect (i.e., when insulin is at basal levels) [2,13]. Si is calculated from two of the model parameters and is defined as fractional disappearance of glucose per insulin concentration unit [2,13].

Several surrogate indices or mathematical models using glucose and insulin levels have been developed as alternative measures of IVGTT. These models include the Homeostatic Model Assessment for Insulin Resistance (HOMA), HOMA 2 (HOMA2), the Quantitative Insulin-sensitivity Check Index (QUICKI), and the Matsuda Index. The simplicity of both the HOMA and the QUICKI models is that they incorporate both fasting insulin and glucose plasma levels [17,18]. Compared to HOMA, HOMA2 considers variations in hepatic and peripheral glucose resistance, increases in the insulin secretion curve for plasma glucose concentrations above 180 mg/dL, and the contribution of circulating proinsulin [19]. The Matsuda Index is a model that uses dynamic glucose and insulin values obtained during a glucose tolerance test [20]. Despite wide use of the models, a universal cutoff value or reference range has not been established for clinical classifications of normal, insulin resistance, prediabetes, and/or T2DM [2]. However, some agreement does exist for the individual models (Table 1). Alternatively, cutoff points and reference ranges are provided by the American Diabetes Association for fasting plasma glucose (FPG) and hemoglobin A1C (HbA1C) (Table 1) [3] given their clinical use to identify and manage glucose dysregulation in persons with and without SCI [1,3,4].

Previous SCI research has evaluated glucose dysregulation using HOMA [26,27,28,29,30,31,32,33,34,35], HOMA2 [36,37,38], Matsuda [28,39,40,41,42], QUICKI [38], HbA1C [43,44,45], and FPG [6,8,35,42,44,46,47]. However, there has been no study or consensus on which insulin resistance/sensitivity index exhibits the best accord with the gold standard IVGTT. Therefore, the objective of this study was to examine the accord among indices of glucose metabolism, including HOMA, HOMA2, the Matsuda Index, QUICKI, HbA1C, and FPG against IVGTT-measured Si in persons with chronic motor complete SCI. We hypothesized superior agreement among QUICKI and Si as measured by IVGTT in persons with chronic motor complete SCI.

## 2. Materials and Methods

### 2.1. Participants, Physical Characteristics, and Body Composition

Participants were consecutively recruited from a SCI registry at the host institution over three years, and through flyers, websites, and local clinics. All participants completed informed consent that was approved by the Institutional Review Board at the host institution. Inclusion criteria included (1) men and women from 18 to 65 years old, with maximum age chosen to avoid any confounding influences of age on body composition; (2) C4-L2 motor complete (American Spinal Injury A & B [48]) individuals; and (3) at least one year post-SCI, as by this time body composition changes stabilize [49]. Only individuals with complete SCI were studied to ensure a homogeneous study sample and to limit the potential influence of incomplete versus complete SCI on body composition [50,51]. Exclusion criteria were as follows: (1) smokers, (2) individuals with excessive alcohol consumption (≥2 drinks/day), (3) those with any known orthopedic limitations and/or uncontrolled spasticity, (3) hypothyroidism, (4) preexisting renal disease or infection/infectious disease, (5) uncontrolled cardiometabolic disorders, (6) deep vein thrombosis or uncontrolled autonomic dysreflexia (hypertensive even after removing noxious stimuli) within the past three months, and/or (7) pressure injuries greater than Grade II.

Prior to measuring height and body mass, each participant was asked to micturate. Height was measured using an anthropometer (Holtain Anthropometry, Middlesex, UK) on the left side after aligning the head, neck, trunk, and lower limbs. Every effort was made to keep the knees in extension [8]. Body mass was measured with a wheelchair scale (PW-630U; Tanita, Arlington Heights, IL, USA). Participants were propelled onto a wheelchair scale with total body weight determined by subtracting the weight of the wheelchair [8]. Body mass index was calculated as weight divided by height squared (kg/m^2^). Total percent body fat, fat free mass, and lean body mass were measured using dual-energy x-ray absorptiometry according to previously published methods [52].

### 2.2. Fasting Blood Plasma and Intravenous Glucose Tolerance Test

Following an overnight 12 h fast, FPG (Wako Chemical USA, Richmond, Virginia, USA) was determined using commercially available colorimetric assays (Thermo DMA, Austin, TX, USA). Fasting insulin was measured with radioimmunoassay single antibody kit (Linco Research, St. Charles, MO, USA). HbA1C was assessed by a VARIANT II TURBO HbA1c testing system (Bio-Rad Laboratories, Hercules, CA, USA).

After the fasting laboratory values were drawn, a standard IVGTT was performed according to previously published methods [15,53]. Briefly, an indwelling catheter with an intravenous saline drip (0.9% NaCl) was placed in an antecubital vein while another intravenous line was placed on a warmed contralateral hand vein. These lines were used to assist with the infusion of glucose/insulin and blood sampling, respectively, throughout the IVGTT. Glucose samples were obtained at −6, −4, −2, 0, 2, 3, 4, 5, 6, 8, 10, 12, 14, 16, 19, 22, 23, 24, 25, 27, 30, 35, 40, 50, 60, 70, 80, 90, 100, 120, 140, 160, and 180 min after the rapid injection of glucose (0.3 g/kg over 30 s at time zero). Twenty minutes following the glucose injection, an insulin bolus (0.02 U/kg of body weight) was injected to determine Si. Heart rate and blood pressure were evaluated at 22, 23, and 24 min of the IVGTT. Si and Sg were computed during the IVGTT using the Minimal Model System (MINMOD Inc., Pasadena, CA, USA.) [13,16].

### 2.3. Calculation of the Insulin Resistance/Sensitivity Indices

HOMA, HOMA2, Matsuda, and QUICKI were calculated according to standard, published methods [1]. Table 1 presents the reference ranges and clinical classifiers with their cutoff values for normal (healthy), prediabetes, T2DM, and/or insulin resistance of the six indices. Clinical classifiers for FPG and HbA1C were operationalized according to the American Diabetes Association (Table 1) [3].

The HOMA was calculated using the following equation:

(1) $$\mathrm{HOMA}=\frac{\mathrm{Fasting}\;\mathrm{Insulin}\;\times \;\mathrm{Fasting}\;\mathrm{Glucose}}{22.5}$$

where fasting glucose and fasting insulin are measured in mg/dL and μU/mL, respectively [18].

HOMA2 was calculated using the open-access HOMA2 calculator downloaded from the Oxford Centre for Diabetes, Endocrinology, and Metabolism at the University of Oxford (https://www.dtu.ox.ac.uk/homacalculator (accessed on 13 November 2020)).

QUICKI was quantified as

(2) $$\mathrm{QUICKI}=\frac{1}{\left(\mathrm{Log}\;\left(\mathrm{Fasting}\;\mathrm{Insulin}\right)+\mathrm{Log}\;\left(\mathrm{Fasting}\;\mathrm{Glucose}\right)\right)}$$

where fasting insulin was measured in μU/ml and fasting glucose was in mg/dL [17]. The Matsuda Index was calculated as follows:

(3) $$\mathrm{Matsuda}\;\mathrm{Index}=\frac{10,000}{\sqrt{(\left(\mathrm{Fasting}\;\mathrm{Glucose}\;\times \;\mathrm{Fasting}\;\mathrm{Insulin}\right)\times \left(\mathrm{Mean}\;\mathrm{Glucose}\;x\;\mathrm{Mean}\;\mathrm{Insulin}\right)})}$$

where fasting and mean insulin were measured in μU/ml and fasting and mean glucose were in mg/dL [20,21]. Mean values of glucose and insulin are derived through measurements taken every 30 min over the three-hour IVGTT [1].

### 2.4. Statistical Analysis

All statistical analyses were performed by using R (R Foundation for Statistical Computing, Vienna, Austria). We chose to only examine Si because it is most frequently evaluated against other surrogate indices in the nondisabled population literature and factors in both plasma glucose and insulin levels during the IVGTT [17,54,55,56]. Continuous values were log-transformed to attenuate the skewness of the data. The log-transformed data were scaled so Si and the indices of HOMA, HOMA2, Matsuda, QUICKI, FPG, and HbA1C had a mean of zero and a standard deviation of one to permit comparison on the same scale. Data were graphically evaluated on a continuous scale and categorically using Bland–Altman plots and dot plot graphics, respectively. Bland–Altman plots (mean of measurement difference ± 2 standard deviation) were used to measure the mean bias (MB) and level of agreement (LOA) against the scaled, log-transformed Si value and the six indices. A dot plot graphic was created using a ggplot for R [57] by graphing the non-scaled, log-transformed Si value by the clinical classifiers (i.e., normal, prediabetes, diabetes, insulin resistant, etc.) for each of the six indices (provides a visual presentation of the agreement). Scatter plots and Kendall rank correlations were used to graph and determine correlations, respectively, between the scaled, log-transformed Si value and the scaled, log-transformed six indices. Linear regression models between the non-scaled, non-log-transformed Si value and each of the clinical classifiers above were performed to calculate R^2^, adjusted R^2^, and the Akaike information criterion (AIC). The AIC was performed to identify the best-fitting model, where smaller values indicate better models with less error. A secondary analysis was performed to convert the trinary classification (e.g., normal, insulin resistance, and diabetes) scales of the FPG, HbA1c, HOMA, and QUICKI indices to a binary scale by collapsing the non-normal (e.g., insulin resistance, prediabetes, T2DM) classifications into a single “abnormal” classification. This allowed for a simple comparison among the six indices. All values are presented as mean and standard deviation and the level of significance was set at alpha < 0.05.

## 3. Results

Twenty-nine participants with chronic motor complete SCI were included in this study. Descriptive statistics on demographic and injury characteristics, body composition, and glucose profiles are presented in Table 2. Table 3 illustrates the total frequency and proportion of the participants with SCI within each clinical classifier according to the six indices’ specific level of classification. In each of the indices, most of the participants were classified as normal (Table 3).

**Figure 1:**
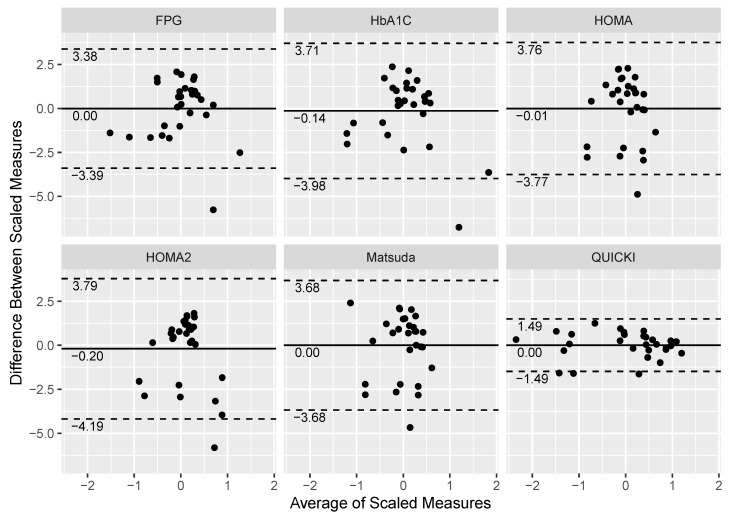
Bland–Altman plots measuring the level of agreement against the scaled, log-transformed Si value and fasting plasma glucose (FPG), hemoglobin A1C (HbA1cC), Homeostatic Model Assessment for Insulin Resistance (HOMA), HOMA2, Matsuda Index, and Quantitative Insulin-sensitivity Check Index (QUICKI). Each data point corresponds to the measurement of 29 participants on Si and the six indices. The solid line represents the mean of the scaled, log-transformed mean difference between two measurements (Si and index), whereas the dashed lines represent the 95% confidence intervals (mean ± 2 standard deviations above and below the mean difference).

**Figure 2:**
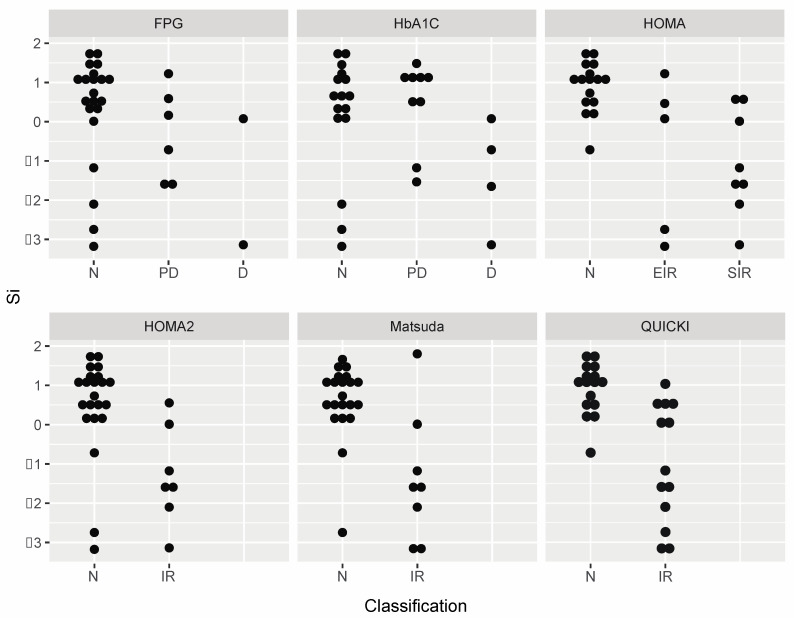
Dot plots of the non-scaled, log-transformed insulin sensitivity (Si) value by the insulin resistance and diabetes classifications of the six indices (n = 29). Fasting plasma glucose (FPG) and Hemoglobin A1C (HbA1C) utilize a normal (N), prediabetes (PD), and diabetes (D) classification scale. The Homeostatic Model Assessment for Insulin Resistance (HOMA) uses a normal (N), early insulin resistance (EIR), and significant insulin resistance (SIR) classification scale, while the Homeostatic Model Assessment 2 for Insulin Resistance (HOMA2) utilizes a normal (N) and insulin resistance (IR) classification scale. The Matsuda Index and Quantitative Insulin-sensitivity Check Index (QUICKI) use a normal (N) and insulin resistance (IR) classification scale.

The degree of concordance between the scaled, log-transformed Si and FPG, HbA1C, HOMA, HOMA2, Matsuda Index, and QUICKI are graphically displayed in Figure 1 and Figure 2. Bland–Altman analysis demonstrated similar MB and LOA between FPG (MB: 0.00, LOA: −3.38, 3.39), HbA1C (MB: −0.14, LOA: −3.98, 3.71), HOMA (MB: −0.01, LOA: −3.77, 3.76), HOMA2 (MB: −0.20, LOA: −4.19, 3.79), and Matsuda Index (MB: 0.00, LOA: −3.68, 3.68) (Figure 1). However, QUICKI had a MB of 0.00 with LOA between −1.49 and 1.49 (Figure 1). Figure 2 shows the agreement of the non-scaled, log-transformed Si value by the clinical classifiers among the six indices. Clustering of the values in the dot plot graphic visually expresses greater accord among QUICKI and HOMA models with Si compared to FPG and HbA1C.

**Figure 3:**
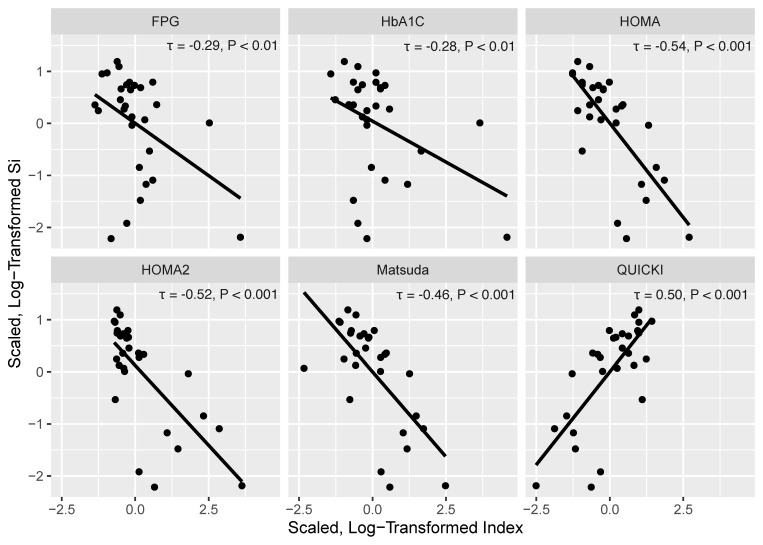
Scatter plots with Kendall rank correlations (τ) of the scaled, log-transformed Si, fasting plasma glucose (FPG), hemoglobin A1C (HbA1C), Homeostatic Model Assessment (HOMA) of Insulin Resistance, HOMA2, Matsuda Index, and Quantitative Insulin-sensitivity Check Index (QUICKI) data.

Figure 3 presents scatter plots with Kendal rank correlation coefficients between Si and the six indices. Scaled, log-transformed HOMA, HOMA2, Matsuda Index, FPG, and HbA1C negatively correlated with Si (τ = −0.28 to −0.54, *p* ≤ 0.01), while the scaled, log-transformed QUICKI positively related to Si (τ = 0.50, *p* < 0.001) (Figure 3). FPG and HbA1C had the smallest tau correlation coefficient, and HOMA and HOMA2 had the highest.

Comparisons using linear regression of the non-scaled, non-log-transformed values from each index to that of Si are found in Table 4. On the trinary scale, QUICKI (adjusted R^2^ = 0.463, AIC = 91.1, *p* = 0.0001) had the greatest agreement and lowest AIC with Si followed by HOMA (adjusted R^2^ = 0.378, AIC = 95.4), HOMA2 (Adjusted R^2^ = 0.256, AIC = 99.7), and the Matsuda Index (Adjusted R^2^ = 0.356, AIC = 95.5) (all *p* ≤ 0.003). FPG and HbA1C predicted approximately 6% (AIC = 107.5) and 10% (AIC = 106.1) of the variance in Si, respectively (*p* > 0.05) (Table 4). Similar patterns and findings were observed with the secondary analysis using the binary scale (Table 4).

## 4. Discussion

The objective of this study was to examine the accord among indices of glucose metabolism with Si as measured by the gold standard IVGTT in persons with chronic motor complete SCI. Our results demonstrate QUICKI has the strongest agreement with Si compared to HOMA, HOMA2, Matsuda Index, FPG, and HbA1C. The weakest agreement with Si was found among FPG and HbA1C, the two most used clinical markers of glucose metabolism [1].

In the present study, QUICKI followed by HOMA had the best agreement with IVGTT-assessed Si in individuals with motor complete SCI. Although QUICKI uses fasting values such as HOMA and HOMA2, it ranked superior to all the indices with regards to its predictive accuracy as evident by the Bland–Altman plots, dot plots, and the adjusted R^2^ and AIC on both the binary and trinary scales. These findings are consistent with previous research in the population without SCI that support QUICKI as the strongest surrogate model to the gold standard markers of glucose metabolism, IVGTT and EHIC [2,54,58,59]. In fact, the significantly higher correlation between QUICKI and EHIC is maintained across nondisabled persons with normal glucose tolerance, impaired glucose tolerance, and T2DM [2,17,55,58]. Katz et al. [17] reported QUICKI was significantly associated to Si of the IVGTT over a range of normal and abnormal insulin sensitivities in nondisabled persons with and without T2DM. In a cohort of 307 men and women, Cheng et al. [54] demonstrated that there were significant correlations between EHIC-measured Si and calculated estimates of QUICKI and HOMA derived from plasma insulin and glucose concentrations of an oral glucose tolerance test (OGTT). The same authors [54] noted QUICKI was the most strongly correlated with EHIC-assessed Si. Chen et al. [55] similarly reported that QUICKI was significantly more accurate than HOMA when compared to Si determined by EHIC in 116 nondisabled persons with and without T2DM. These findings illustrate the superiority of QUICKI to screen for and diagnose disorders of glucose regulation in persons with SCI when IVGTT is unavailable.

In the nondisabled population, QUICKI has been reported to have less variability, high reliability, and high discriminant power as assessed by the discriminatory ratio (DR) [2]. In a study of 152 healthy, obese, nondiabetic, and T2DM nondisabled subjects, Mather et al. [60] reported a significantly greater discriminatory power in QUICKI (DR = 10.2) than the HOMA model (DR = 3.4). Additionally, QUICKI has considerably less variability (coefficient of variance [CV] = 3.9%) than HOMA (CV = 26.7%) [1,61,62]. Henriquez et al. [63] compared the variability among HOMA, HOMA2, and QUICKI in 80 healthy nondisabled subjects and reported that the CV was worse for HOMA and HOMA2 (>10%) and superior for QUICKI (<3%). This difference in variability is likely due to the inherent normalization of QUICKI values. Logarithmic transformations are commonly performed to make patterns in data more readily interpretable. This is completed by reducing the potential influence of extreme value distributions on the results, thereby approximating a normal distribution. Collectively, the superiority of QUICKI relative to other markers is likely a result of the built-in logarithmic function that normalizes the distribution of fasting glucose and insulin values among persons with and without SCI.

Studies have reported that the performance of HOMA can be improved by logarithmically transforming its values, thereby converting the nonlinear hyperbolic relationship between HOMA and Si (measured by EHIC or IVGTT) into a linear relationship [2,17,56,59]. Both Otten et al. [59] and Mather et al. [60] have reported that after logarithmically transforming HOMA values, the correlation between HOMA and Si as measured by EHIC becomes comparable to that of QUICKI and EHIC. In the current study, HOMA had the next best agreement with Si of the IVGTT after QUICKI in persons with SCI, potentially because of the logarithmic transformation of the HOMA values.

FPG and HbA1C were found to have the worst accord with Si of the IVGTT compared to the other indices. Similar findings have been previously reported in persons with and without SCI [64,65,66,67,68,69]. Both Duckworth et al. [70] and Bauman et al. [71] have reported normal FPG levels in persons with SCI previously diagnosed with impaired glucose tolerance and T2DM. Stillman et al. [72] identified 47% of individuals with SCI that had either an elevated HbA1C or 2 h glucose level after a 75 g OGTT but found only 22% of the study participants had both present. Similarly, in 95 persons with acute SCI, Solinsky and colleagues [36] recently identified insulin resistance in 12.5% of the cohort using elevated FPG as a criterion but in 33.3% when using HOMA2 criteria. The discord between IVGTT-measured Si and both FPG and HbA1C may be due to the methodical differences by which the latter two markers define abnormal glucose metabolism [69,73]. Unlike the other indices, FPG and HbA1C do not account for insulin levels or the acute insulin-induced changes in glucose [69,73,74]. Rather, FPG simply measures glucose in blood plasma following a minimum 8 h fast [4]. HbA1C reflects long-term glycemic control and estimates protein glycation instead of measuring blood glucose directly [69,73,74]. These findings are of importance given the risk and occurrence of glucose dysregulation after SCI [1,4] and how FPG and HbA1C are routinely used to identify and manage glucose metabolism in this population [4]. It is therefore likely that FPG and HbA1C, if used diagnostically, are underestimating prediabetes and T2DM in persons with SCI. The health, function, and economic burden associated with poor screening methods is of significant concern given the already large proportion of persons with SCI living with metabolic comorbidities [11]. 

It is important to note that research has shown that the diagnostic utility of FPG and HbA1C can be improved when used in combination rather than in isolation [75,76,77]. In 136 persons with T2DM, Lorenzo et al. [75] reported that the detection of T2DM increased to 52% when FPG and HbA1C were used in combination versus the 32% for HbA1C and 45% for FPG when they were used alone. Moreover, Yan et al. [77] demonstrated that the sensitivity (67–71%) and specificity (55–80%) of HbA1C significantly improved across various age groups when combined with FPG (80–82% sensitivity, 99–100% specificity). In the present study, FPG and HbA1C classified 3.4% and 10.3% of the study participants with diabetes, respectively. QUICKI, however, was able to identify diabetes in 17.2% of the participants. To the authors’ knowledge, no research has used both FPG and HbA1C in combination to evaluate T2DM risk in SCI. Collectively, these findings suggest that the combination of HbA1C and FPG (in the absence of fasting insulin to calculate QUICKI) may be more clinically useful in screening persons with SCI rather than relying on a single test as the current Clinical Practice Guidelines for Identification and Management of Cardiometabolic Risk after SCI suggest [4].

### Study Limitations

This study is not without limitations. First, we did not recruit a control group without SCI, and our sample size was small. The limited sample size may have resulted in a Type 2 error, although several findings were significant. Second, our study included only one of the gold standard measurements of Si, the IVGTT, and did not include assessment from the similar EHIC or OGTT. As previously noted, EHIC has inherent limitations (i.e., safety), making its use a risk for persons with SCI. It also has been reported that there is some discord between OGTT and IVGTT in the identification of prediabetes but not in healthy nondisabled persons or those with T2DM [78]. Third, many of the methods involved in our study rely on fasting values of insulin, but the biological variability of insulin levels provides a source of variation due to insulin’s short half-life, the cyclicity of insulin secretion, and the rapid responsiveness to small changes in hormones and metabolism [18,59,60]. Fourth, the cutoff values defining normal, prediabetes, T2DM, and/or insulin resistance are based on the population without SCI. It is currently unknown if these cutoff values are appropriate in persons with SCI. Additionally, HOMA2 does not have an accepted reference range to define normal insulin sensitivity versus insulin resistance compared to other models [1]. Rather, this study used the cutoff value described in Gordon et al. [1], which suggests an optimal HOMA2 cutoff value of 1.4 derived from independent testing based in several countries (Kuwait [22], Turkey [23], Iran [24], and Brazil [25]). Fifth, we did not account for race or gender because of a limited sample size to investigate their respective influences. This research should, however, serve as a first step to future studies to investigate the influence of race and gender on glucose metabolism in SCI. Lastly, we were unable to calculate the coefficient of variation or discriminatory power because we did not have repeated measures taken several hours apart or on separate days. However, both binary and trinary scales and the AIC of the linear regression, along with the Bland–Altman and dot plots, consistently supported the use of QUICKI.

## 5. Conclusions

This study demonstrates a difference between the performance of clinical versus non-clinical indices of glucose metabolism. Although HbA1C and FPG are more commonly used for the identification and management of glucose dysregulation, our results indicate the superiority of QUICKI. This superiority is likely given QUICKI’s built-in log-transformation that provides a stronger linear correlation with IVGTT-measured Si [17,18,79]. FPG and HbA1C had the poorest agreement with the IVGTT; as a result, these markers should be used in combination to improve the diagnostic precision when QUICKI is unavailable. Future research is required to determine the agreement between the OGTT and IVGTT and evidence-based, SCI-specific cutoff values for QUICKI.

## Figures and Tables

**Table 1:** Clinical classifiers and their reference ranges for disorders of glucose dysregulation.

Index	Reference Ranges
Fasting plasma glucose [1,3]	Normal: <100 mg/dL
	Prediabetes: 100–125 mg/dL
	T2DM: ≥126 mg/dL
Hemoglobin A1C [1,3]	Normal: <5.7%
	Prediabetes: 5.7–6.4%
	T2DM: ≥6.5%
Homeostatic Model Assessment of IR [1,18]	Normal: ≤1.6
	Early IR: 1.7–2.4
	Significant IR: ≥2.5
Homeostatic Model Assessment 2 of IR [1] *	Normal: < 1.4
	IR: ≥1.4
Matsuda Index [1,20,21]	Normal: >2.5
	IR: ≤2.5
Quantitative Insulin-sensitivity Check Index [1,17]	Normal: >0.339
	IR: ≤0.339

IR, Insulin resistance; T2DM, type 2 diabetes mellitus. * There are currently no universal cut-off points to define normal versus abnormal HOMA2 values, but several studies examining the general adult populations of Kuwait [22], Turkey [23], Iran, [24], and Brazil [25] have independently reported that a HOMA2 value of 1.4 is the optimal cut-off point to identify persons with insulin resistance.

**Table 2:** Demographic, body composition, and glucose metabolism data in persons with chronic motor complete SCI (*n* = 29).

Demographic and Injury Characteristics	
Age (years)	42.2 (11.4)
Sex (% male)	79.3%
Body mass index (kg/m^2^)	28.6 (6.4)
Body weight (kg)	87.8 (22.9)
Height (m)	1.8 (0.09)
Time since injury (years)	14.5 (11.6)
Level of injury	C4-T10
Injury severity (ASIA Impairment Scale %A/%B)	(86.2/13.8%)
**Body Composition**	
Fat free mass (kg)	52.8 (11.6)
Lean body mass (kg)	48.7 (10.7)
Total body fat (%)	40.4 (8.9)
**Glucose Metabolism**	
Insulin sensitivity (min^−1^/(µU/mL^−1^) × 10^−4^)	2.3 (1.8)
Glucose effectiveness (min^−1^)	0.02 (0.01)
Fasting plasma insulin (uU/L)	9.7 (9.0)
Fasting plasma glucose (mg/dL)	95.4 (28.4)
Hemoglobin A1C (%)	5.7 (0.7)
Homeostatic Model Assessment for Insulin Resistance	2.7 (3.8)
Homeostatic Model Assessment 2 for Insulin Resistance	1.3 (1.2)
Matsuda Index	6.9 (4.5)
Quantitative Insulin-sensitivity Check Index	0.36 (0.04)
Data presented as mean (SD).	

**Table 3:** Classification of glucose metabolism by index (*n* = 29).

	*n* (%)
**Fasting Plasma Glucose (mg/dL)**	
Normal	22 (75.9%)
Prediabetes	6 (20.7%)
Diabetes	1 (3.4%)
**Hemoglobin A1C (%)**	
Normal	18 (62.1%)
Prediabetes	8 (27.6%)
Diabetes	3 (10.3%)
**Homeostatic Model Assessment for Insulin Resistance**	
Normal	18 (62.1%)
Early Insulin Resistance	7 (24.1%)
Significant Insulin Resistance	4 (13.8%)
**Homeostatic Model Assessment 2 for Insulin Resistance**	
Normal	22 (75.9%)
Insulin Resistance	7 (24.1%)
**Matsuda Index**	
Normal	22 (75.9%)
Insulin Resistance	7 (24.1%)
**Quantitative Insulin-sensitivity Check Index**	
Normal	18 (62.1%)
Insulin Resistance	11 (37.9%)

**Table 4:** Linear regression models between the non-scaled, non-log-transformed Si value and each of the classifiers from the six indices.

Trinary Scale				
	R^2^	Adjusted R^2^	Akaike Information Criterion	*p*-Value
Fasting plasma glucose	0.124	0.056	107.5	0.1799
Hemoglobin A1C	0.164	0.100	106.1	0.0975
HOMA	0.422	0.378	95.4	0.0008
HOMA2	0.282	0.256	99.7	0.0030
Matsuda Index	0.379	0.356	95.5	0.0004
QUICKI	0.501	0.463	91.1	0.0001
Binary Scale *				
	**R^2^**	**Adjusted R^2^**	**Akaike Information Criterion**	***p*-Value**
Fasting plasma glucose	0.087	0.053	106.7	0.1206
Hemoglobin A1C	0.009	−0.027	109.1	0.6175
HOMA	0.420	0.398	93.5	0.0001
HOMA2	0.282	0.256	99.7	0.0030
Matsuda Index	0.379	0.356	95.5	0.0004
QUICKI	0.501	0.463	91.1	0.0001

HOMA, Homeostatic Model Assessment for Insulin Resistance; HOMA2, Homeostatic Model Assessment 2 for Insulin Resistance; QUICKI, Quantitative Insulin-sensitivity Check Index * Binary scale reflects the conversion of the trinary classification scales (e.g., normal, insulin resistance, diabetes) of the FPG, HbA1c, and HOMA indices to a binary scale by collapsing the non-normal (e.g., insulin resistance, prediabetes, T2DM) classifications into a single “abnormal” classification.

## Data Availability

The dataset generated and/or analyzed during the study are available from the corresponding author upon reasonable request, given approval is provided by University’s Institutional Review Board.

## Abbreviations

FPG	Fasting plasma glucose
CV	Coefficient of variance
DR	Discriminatory ratio
EHIC	Euglycemic hyperinsulinemic clamp
HbA1C	Hemoglobin A1C
HOMA	Homeostatic Model Assessment of Insulin Resistance
HOMA2	Homeostatic Model Assessment 2 of Insulin Resistance
IVGTT	Intravenous glucose tolerance test
OGTT	Oral glucose tolerance test
QUICKI	Quantitative Insulin-sensitivity Check Index
SCI	Spinal cord injury
Sg	Glucose effectiveness
Si	Insulin sensitivity
T2DM	Type 2 diabetes mellitus
